# Occurrence and Multi-Locus Genotyping of *Enterocytozoon bieneusi* in Black Goats from Fujian Province, China

**DOI:** 10.3390/pathogens15030299

**Published:** 2026-03-10

**Authors:** Kai Hu, Zhong-Yang Chen, Yanlong Gu, Sheng-Jie Tang, Peng-Fei Fu, Dong-Hui Zhou

**Affiliations:** 1 Key Laboratory of Fujian-Taiwan Animal Pathogen Biology, College of Animal Sciences, Fujian Agriculture and Forestry University, Fuzhou 350002, China; 2 Key Laboratory of Animal Pathogen Infection and Immunology of Fujian Province, College of Animal Sciences, Fujian Agriculture and Forestry University, Fuzhou 350002, China

**Keywords:** *Enterocytozoon bieneusi*, black goats, genotype, Fujian province

## Abstract

*Enterocytozoon bieneusi* is an important zoonotic intestinal protozoon causing diarrhea in humans and animals, threatening public health and livestock farming. This study aimed to investigate the infection and genotype distribution of *E. bieneusi* in black goats in Fujian, southeastern China. A total of 539 fecal samples were collected from nine regions. *E. bieneusi* was detected by nested PCR and sequencing targeting the ITS locus, and genotyped using four microsatellites and one minisatellite. The overall infection rate was 7.79%, with 42 positive samples. Eight genotypes were identified, including seven known genotypes and one novel genotype FJG-1. Ten multilocus genotypes (MLGs) were obtained, and all isolates belonged to Group 2. The infection rate differed significantly among regions (*p* < 0.01), but not among ages or genders (*p* > 0.05). This is the first molecular epidemiological survey of *E. bieneusi* in black goats in Fujian. The results enrich the genotype database and provide data for formulating regional prevention and control strategies against this pathogen.

## 1. Introduction

*Enterocytozoon bieneusi* is an important zoonotic pathogen with significant public health implications [1,2,3]. As an obligate intracellular pathogen, it has an extremely broad host range, encompassing humans, various mammals, birds, insects, and fish [4,5,6,7]. Microsporidiosis is an intestinal infectious disease caused by pathogens of the genus Microsporidia and is one of the important protozoan causes of zoonotic diarrhea. Among human microsporidiosis cases, *E. bieneusi* is the most common pathogen, accounting for approximately 90% of confirmed cases [5,6]. This pathogen specifically infects small intestinal epithelial cells, which can lead to typical histopathological changes such as villus atrophy, epithelial cell degeneration and necrosis, and subsequently cause chronic diarrhea, malabsorption and a series of gastrointestinal functional disorders [8]. It is worth noting that it not only causes severe symptoms in people with weakened immune systems, such as AIDS patients, organ transplant recipients and cancer patients, but also triggers asymptomatic or self-limiting diarrhea in people with normal immune function [9]. The existence of asymptomatic infections further highlights its potential role in the epidemiology of microbial transmission [10].

Despite the fact that they were once regarded as primitive protozoa, phylogenetic analyses have placed them within the fungal kingdom, in close association with the *Cryptomycota* [11]. This reclassification is supported by multiple lines of evidence: phylogenetic analyses of α- and β-tubulin, mitochondrial-like HSP70, and other genes have demonstrated their relationship to fungi [12]; genomic data from Encephalitozoon cuniculi also support this conclusion [13,14]. Furthermore, the presence of relict mitochondrial genes in the nuclear genome, the existence of mitosomes and Golgi-like membrane structures, as well as their unique life cycle characteristics, provide further support for this classification. To date, more than 860 unique genotypes of *E. bieneusi* have been reported globally, distributed across 50 countries and involving 210 host species. Among them, 126 genotypes have been found only in human cases (human-specific), 614 have been detected only in animal hosts (animal-specific), and another 58 genotypes have been jointly identified in both human and animal hosts, suggesting a clear risk of zoonotic transmission [15]. All the genotypes identified so far have been systematically classified into 13 evolutionary groups (Groups). Among them, Group 1 and Group 2 have concentrated the vast majority of human infection cases and zoonotic-related genotypes, highlighting their public health significance as important clusters for the transmission of zoonotic diseases [16,17,18].

Studies have shown that goats are important natural hosts of *E. bieneusi* [19]. China has accumulated systematic and high-quality data in the field of molecular epidemiology of *E. bieneusi* from cattle and goats, forming a regional research advantage with international influence [20,21,22]. However, there is a lack of molecular epidemiological data on *E. bieneusi* in black goats in Fujian province (southeastern China), and the geographical distribution, genetic diversity and cross-host transmission potential of this pathogen in local black goat populations remain unclear. In addition, the genotype distribution map of *E. bieneusi* in goats in southeastern China is still incomplete, which restricts the regional risk assessment and precise prevention and control of this zoonotic pathogen.

The multilocus sequence typing (MLST) technology based on microsatellite (MS1, MS3, MS7) and minisatellite (MS4) loci has become an efficient molecular tool for analyzing the genetic diversity, population structure and transmission chain traceability of *E. bieneusi*, effectively compensating for the limitations of single-locus internal transcribed spacer (ITS) typing in terms of resolution and evolutionary inference ability [23]. In this study, fecal samples from black goat populations in nine prefecture-level cities in Fujian province were systematically collected, and nested PCR targeting the ITS gene was used to screen for *E. bieneusi* infection; MLST analysis was further conducted on all positive samples to comprehensively assess their genotype diversity, geographical distribution pattern and cross-host transmission potential. This study is the first to systematically reveal the epidemiological characteristics and molecular epidemiological map of *E. bieneusi* in black goats in Fujian province. The obtained data will provide key scientific support for regional risk assessment, formulation of precise prevention and control strategies, and the construction of a joint prevention and control mechanism for zoonotic diseases, effectively serving the dual goals of high-quality development of animal husbandry and public health security.

## 2. Materials and Methods

### 2.1. Study Areas and Sample Collection

**Figure 1:**
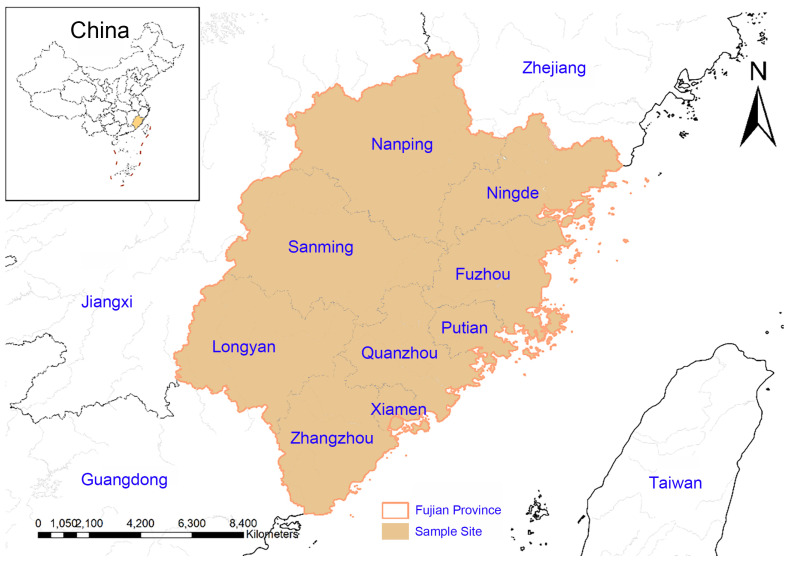
Distribution of sampling sites in Fujian.

From September to November 2023, a total of 539 fecal samples from black goats were randomly collected from goat farms in nine regions of Fujian province (for collection details, refer to Table 1 and Figure 1), including 161 from rams and 378 from ewes. In each of the nine prefecture-level cities, one farm was selected for sampling. The number of fecal samples collected per farm ranged from 16 to 82, depending on the scale of each operation, and the detailed sample sizes are presented in Table 1. Among the 539 samples, 228 were from goats under 1 year of age, 181 from those aged 1–2 years, 97 from the 2–3 year group, and 33 from goats over 3 years old. All black goats in this study were indoor group-housed in enclosed pens and did not have access to pasture or open watercourses, which minimized the risk of environmental exposure to potential pathogens. Fecal samples were collected directly from the rectum of each animal using sterile gloves to ensure that specimens were not contaminated by the environment and accurately reflected the individual infection status. Samples were then transported to the laboratory under refrigeration and stored at −80 °C until further analysis. This research was approved and conducted under the supervision of the Ethics Committee of Fujian Agriculture and Forestry University (Approval Number: PZCASFAFU22022, Approval Date: 15 March 2022).

### 2.2. Genomic DNA Extraction

Total genomic DNA was extracted from 200 mg of each fecal sample using the Genomic DNA Extraction Kit (TianGen Biotech, Beijing, China). All DNA samples were stored at −20 °C until further analysis.

### 2.3. PCR Amplification and Electrophoresis

In this study, positive samples for *E. bieneusi* were genotyped using the multilocus sequence typing (MLST) scheme established by Y.Y. Feng et al. [24]. This scheme is based on three microsatellite loci (MS1, MS3, MS7) and one minisatellite locus (MS4) (Primer information is provided in Table S1). Nested PCR was performed for each of the four loci. The reaction mixture for both PCR rounds was identical. The PCR reaction mixture (25 µL) contained 2.5 µL of 10× PCR Buffer, 2 µL of dNTPs, 0.2 µL of Ex Taq DNA polymerase, 0.25 µL of each forward and reverse primer, and 1.54 µL of MgCl_2_, with the remaining volume made up with ddH_2_O. All reagents were purchased from TaKaRa (Dalian, China). The difference between the two PCR rounds lay in the template: the first round used genomic DNA extracted from the samples as the template, while the second round used the amplification products from the first round as the template. The amplification conditions, except for the annealing temperature, which varied, were as follows: initial denaturation at 94 °C for 5 min, followed by 35 cycles of denaturation at 94 °C for 45 s, annealing (Annealing temperatures are provided in Table S1) for 30 s, and extension at 72 °C for 40 s, with a final extension at 72 °C for 10 min. During each PCR amplification, positive and negative control samples are added to ensure the reliability of the results. Positive control DNA was obtained from a previously confirmed *E. bieneusi*-positive fecal sample stored in our laboratory. Negative control (no-template control) consisted of nuclease-free water instead of DNA template. Both controls were included in each PCR run to monitor for contamination and amplification efficiency. A 5 µL aliquot of each PCR product was mixed with 1 µL of 6× Loading Buffer, loaded into the sample wells of a 1% agarose gel containing nucleic acid dye, with the Takara DL2000 DNA Marker (Takara, Dalian, China) used as the molecular weight standard. Electrophoresis was performed in TAE buffer (Shanghai Tongsheng Biotechnology Co., Ltd., Shanghai, China) at 125 V for 30 min. Following electrophoresis, the gel was visualized and documented under UV light using a gel imaging system.

### 2.4. Sequencing and Phylogenetic Analysis

All second-round PCR products corresponding to the expected band size were sent to Sangon Biotech (Shanghai) Co., Ltd. (Shanghai, China) for bidirectional sequencing. The returned sequencing results were assembled and corrected using the bioinformatics software DNAstar 7.1. The assembled sequences were then compared online via the NCBI database to determine the genotype of *E. bieneusi* and analyze its polymorphism. Phylogenetic analysis was performed using MEGA 11 software. Sequence alignment was conducted with the Clustal W method, and a phylogenetic tree was constructed using the neighbor-joining (NJ) method with 1000 bootstrap replications. The novel genotype sequences identified in this study have been submitted to the NCBI GenBank database.

### 2.5. Statistical Analysis

Statistical analysis of the *E. bieneusi* infection rates in black goats across different regions, age groups, and sexes was performed using SPSS Statistics 26.0. Differences in infection rates among groups were compared using the χ2 test. A *p*-value < 0.05 was considered statistically significant.

## 3. Results

### 3.1. Detection of the Infection Rate of E. bieneusi

The ITS gene of *E. bieneusi* was used as the detection target to test 539 black goat fecal samples collected from nine regions in Fujian province. A total of 42 positive samples were identified by nested PCR, with an overall positive rate of 7.79%.

### 3.2. Infection Rates and Genotype Prevalence of E. bieneusi in Black Goats from Different Regions of Fujian Province

There were significant differences in the infection rates and genotype prevalence of *E. bieneusi* in black goats across different regions of Fujian province, with the infection rates ranging from 0% to 22.85%. Among these regions, Nanping City (22.85%, 16/70) had the highest infection rate, followed by Zhangzhou City (18.29%, 15/82). In contrast, no positive samples were detected in Quanzhou City (0%, 0/39) and Ningde City (0%, 0/57) (Table 2). The highest number of infected genotypes (6 types) was detected in Zhangzhou. Statistical analysis showed that there were significant differences in the infection rate and genotype prevalence among various regions in Fujian province (*p* < 0.01). Positive samples were detected in 7 out of the 9 farms surveyed, indicating that *E. bieneusi* infection is endemic across multiple regions of Fujian Province rather than confined to a single farm or localized outbreaks. The distribution of positive samples across farms was uneven, with some farms (Zhangzhou) harboring multiple genotypes, suggesting complex transmission dynamics within herds.

### 3.3. Infection Rates and Genotype Prevalence of E. bieneusi in Black Goats of Different Ages in Fujian Province

The infection rates of *E. bieneusi* in black goats of different age groups in Fujian province ranged from 0 to 8.40%. Specifically, the positive rate was 7.09% (20/228) in black goats aged ≤1 year, 8.84% (16/181) in those aged 1–2 years, and 6.19% (6/97) in those aged 2–3 years, while all individuals aged ≥3 years tested negative (0%, 0/33) (Table 3). Statistical analysis revealed no significant difference in the positive rates of *E. bieneusi* among different age groups (*p* > 0.05), indicating that age may not be a key factor influencing the infection rate of *E. bieneusi*.

### 3.4. Infection Rates and Genotype Prevalence of E. bieneusi in Black Goats of Different Genders in Fujian Province

The gender-specific infection rate of *E. bieneusi* in black goats in Fujian province ranged from 7.14% to 9.32%. Specifically, the infection rate was 9.32% (15/161) in rams and 7.14% (27/378) in ewes (Table 4). No obvious differences were observed in the infected genotypes between rams and ewes. Statistical analysis revealed no significant difference in the positive rate of *E. bieneusi* between different genders (*p* > 0.05), indicating that gender is not a key factor influencing the *E. bieneusi* infection rate in black goats.

### 3.5. Genotype Identification of E. bieneusi

**Figure 2:**
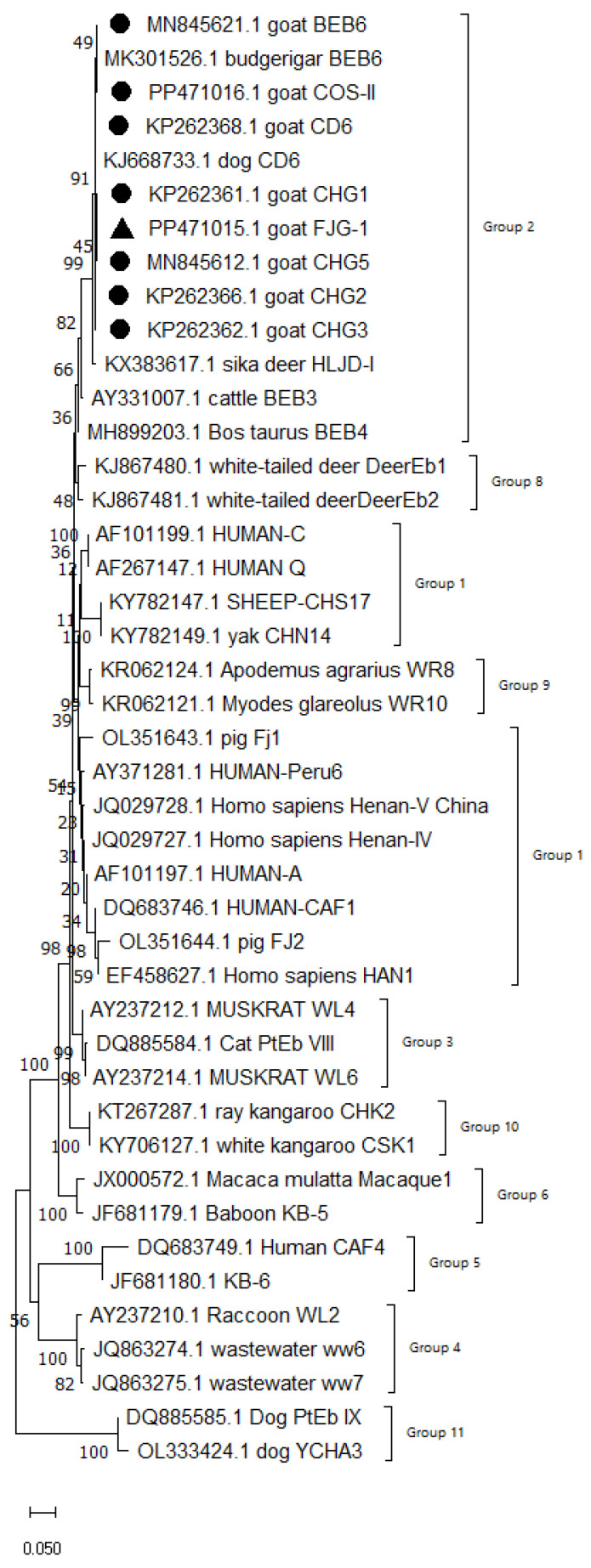
The phylogenetic evolutionary tree of *E. bieneusi.* ● represents the genotypes identified in this experiment; ▲ represents new genotypes.

The ITS locus of these 42 positive samples were all successfully amplified. After alignment, trimming and splicing of the amplified sequences, BLAST(v.2.17.0) search and analysis were performed. The results revealed the identification of eight genotypes, namely CHG1, CHG2, CHG3, CHG5, BEB6, CD6, COS-II and FJG-1. Phylogenetic analysis of the positive sample sequences against reference sequences demonstrated that all eight genotypes belonged to Group two (Figure 2), which carries a high zoonotic risk.

The CD6 genotype accounted for 47.6% of all positive samples (20/42) and was distributed in black goats of different infection ages and genders, making it the dominant genotype of *E. bieneusi* infection in black goats in Fujian province, followed by CHG3 (8 samples), accounting for 19% (8/42).

Two representative DNA sequences of the ITS locus were obtained from the 42 positive samples and submitted to the GenBank database (IDs: PP471015-PP471016).

Phylogenetic evolution of the ITS locus was analyzed using MEGA 11 software, followed by sequence alignment via the Clustal W method, and a phylogenetic tree was finally constructed using the Neighbor-Joining (NJ) method (Figure 1).

### 3.6. MLST of E. bieneusi

A total of 42 positive samples were identified by detection at the IST locus. Among these, twelve samples yielded positive amplification at the MS1 locus with an amplified fragment length of 675 bp, corresponding to a positive rate of 2.22% (12/539). Additionally, seventeen samples were positively amplified at the MS4 locus with an amplified fragment length of 885 bp, representing a positive rate of 3.15% (17/539), and eighteen samples showed positive amplification at the MS7 locus with an amplified fragment length of 471 bp, with a positive rate of 3.34% (18/539).

A total of ten novel multilocus genotypes (MLGs), designated as MLG I to MLG X (Table 5), were successfully identified at the MS1, MS4 and MS7 loci, which confirmed that *E. bieneusi* infecting black goats exhibits the characteristic of molecular genetic differentiation.

## 4. Discussion

The results of this study show that the overall infection rate of Enterocytozoon bieneusi in the investigated black goat population in Fujian province was 7.79%, which was lower than that in Yunnan (10.59%), Heilongjiang (21.8%), Chongqing (62.5%), Henan (32.9%), Shaanxi (47.8%), Iran (10.6%), and Sweden (68.0%) [25]. It should be noted that comparisons between this study and those conducted in other regions are subject to certain limitations: cross-regional comparisons did not fully account for methodological differences, including potential confounding factors such as variations in the age composition of animals, types of farming models, and sampling seasons across different studies. These factors may have biased the comparison results of prevalence rates and genotype distributions among regions. Among the nine prefecture-level cities covered, positive samples were detected in seven regions, and the infection rates showed significant regional differences: the highest was in Nanping City (22.85%, 16/70), followed by Zhangzhou City (18.29%, 15/82), Xiamen City (6.25%, 1/16), Longyan City (5.26%, 4/76), Putian City (4.84%, 3/62), Fuzhou City (3.13%, 2/64), and Sanming City (1.36%, 1/73); no positive samples were detected in Quanzhou City and Ningde City (0/39 and 0/57). This variation may be attributed to differences in farm management practices, stocking density, and biosecurity measures. Nanping and Zhangzhou have larger-scale goat farming operations with higher animal densities, which may facilitate fecal–oral transmission of *E. bieneusi*. In contrast, Quanzhou and Ningde are characterized by smaller-scale, scattered farming systems with limited animal movement, potentially reducing pathogen exposure. Additionally, differences in sampling season, climatic conditions, and geographical factors may also influence the detection rates, as suggested by previous studies [26].

In the age distribution analysis of all *E. bieneusi*-positive samples in this study, no statistically significant difference in infection rates was found among groups, indicating that age is not a risk determinant for *E. bieneusi* infection in this population. Additionally, the gender analysis results showed that gender had no significant impact on the risk of *E. bieneusi* infection in black goats in Fujian province. This finding can provide an epidemiological basis for optimizing the population structure and formulating health management strategies in large-scale breeding.

A total of eight genotypes were detected in the black goats in this study, including seven known genotypes (CHG1, CHG2, CHG3, CHG5, BEB6, CD6 and COS-II) and one new genotype (FJG-1). Sequence analysis revealed that the novel genotype FJG-1 (PP471015) was most closely related to COS-II (PP471016), differing by only four single-nucleotide polymorphisms (SNPs) in the complete ITS region: T→C at position 47, T→G at position 111, T→C at position 169, and A→G at position 208. Compared with other known genotypes, FJG-1 exhibited five SNPs with CHG1 (KP262361) and CHG3 (KP262362), six SNPs with CHG2 (KP262366), CD6 (KP262368) and CHG5 (MN845612), and seven SNPs with BEB6 (MN845621). All variations were single base substitutions without insertions or deletions (indels). The high sequence similarity between FJG-1 and COS-II indicates that they belong to the same evolutionary clade, while the unique SNP profile supports the identification of FJG-1 as a novel *E. bieneusi* genotype. Among them, CD6 is the dominant genotype of *E. bieneusi* infection in Fujian black goats. This might be because the CD6 genotype belongs to the Group 2, and cattle and sheep are the main hosts for the infection of the Group 2 genotypes [27]. CHG2, CHG3 and CHG5 are the most common genotypes in goats and have been found in almost all studies on goats in China [17,18]. Notably, these genotypes were discovered in humans, goats and geese in Hainan, China, especially the CHG5 genotype, which was also detected in cattle and rodents in Hainan, indicating that these genotypes pose a potential zoonotic risk [28,29,30]. BEB6 is a zoonotic genotype with a wide host range, including humans [31], non-human primates [32], cattle [33], deer [34], golden takins [35], captive wildlife [36], cats [37] and birds [38].

Further phylogenetic analysis revealed that all the eight genotypes detected in this study belong to Group two. Among these, CHG2, CHG3, CHG5, and BEB6 are established zoonotic genotypes, while CHG1, COS-II, and the novel genotype FJG-1, having no reported human infections to date, are classified as potential zoonotic risk genotypes. Therefore, the black goat farm needs to enhance the prevention and control of *E. bieneusi* and raise the awareness of the breeding personnel regarding their own health protection.

According to existing reports, multiple haplotype differences at the same ITS locus have been found in goats. In this study, positive samples were detected at the MS1, MS4, and MS7 loci, but amplification failed for all ITS positive samples at the MS3 locus. ITS genotype identification revealed that it has host adaptation characteristics. Therefore, the MLST loci established might not have corresponding loci or have extremely low expression levels in some host-specific evolutionary groups [39]. This could be the reason why certain loci (such as MS3) in this study did not yield the target bands even after multiple amplifications. In this study, 10 samples were successfully amplified at 3 polymorphic loci, and 10 MLGs were identified by MLST analysis.

## 5. Conclusions

This study revealed that the prevalence of *E. bieneusi* in black goats from nine cities in Fujian province was 7.79%. There were significant statistical differences among regions, while no significant differences were found in terms of age and gender. Through PCR amplification of the ITS locus, a total of eight genotypes were detected, including seven known genotypes (CHG1, CHG2, CHG3, CHG5, BEB6, CD6, and COS-II) and one new genotype (FJG-1). Among them, CD6 was the predominant genotype. Phylogenetic analysis indicated that all eight genotypes detected in this study belonged to Group two, posing a potential zoonotic risk. MLST analysis showed that ten *E. bieneusi* multi-locus positive samples formed ten MLGs, and the same ITS locus exhibited multiple genetic diversities. These data provide support for the epidemiology and control of *E. bieneusi* infection in black goats in Fujian province.

## Figures and Tables

**Table 1:** Sampling information of Fujian region.

Region	Gender	Age					Summation	Total
		0–1 Year	1–2 Years	2–3 Years	3–4 Years	4–5 Years		
Putian	Male	10	0	7	2		62	539
	Female	10	7	8	16	2		
Sanming	Male	6	10	2			70	
	Female	17	30	4	1			
Quanzhou	Male	5	1	1			39	
	Female	10	6	11	5			
Zhangzhou	Male	14	10	1			82	
	Female	11	32	13	1			
Longyan	Male	21	7	2			76	
	Female	21	14	11				
Xiamen	Male	6	3				16	
	Female	1	6					
Nanping	Male	12	11	1			70	
	Female	18	18	9	1			
Ningde	Male	17	2				57	
	Female	27	9	1		1		
Fuzhou	Male	10					67	
	Female	12	15	26	3	1		

**Table 2:** *E. bieneusi* genotypes of different areas in Fujian province.

District	Sample Number	Infection Rate (95% CI)	Assemblages
Putian	62	3 (4.84%, 1.01–13.50%)	CD6(3)
Sanming	73	1 (1.36%, 0.03–0.74%)	CHG2(1)
Quanzhou	39	0 (0%, 0.00–9.03%)	
Zhangzhou	82	15 (18.29%, 10.62–28.37%)	CHG2(2), CHG3(6), CHG5(1), BEB6(3), CD6(2), FJG-1(1)
Longyan	76	4 (5.26%, 1.45–12.93%)	CHG1(1), CHG5(2), FJG-1(1)
Xiamen	16	1 (6.25%, 0.16–30.20%)	BEB6(1)
Nanping	70	16 (22.85%, 13.67–34.45%)	CD6(15), COS-II(1)
Ningde	57	0 (0%, 0.00–6.27%)	
Fuzhou	64	2 (3.13%, 0.38–10.84%)	CHG3(2)
Total	539	42 (7.79%, 5.67–10.39%)	CHG1(1), CHG2(3), CHG3(8), CHG5(3), BEB6(4), CD6(20), COS-II(1), FJG-1(2)

**Table 3:** *E. bieneusi* genotypes of different ages in Fujian province.

Age	Sample Number	Infection Rate (95% CI)	Assemblages
≤1 year	228	20 (7.09%, 5.44–13.22%)	CHG1(1), CHG3(3), CHG5(3), BEB6(4), CD6(8), FJG-1(1)
1–2 years	181	16 (8.84%, 5.14–13.06%)	CHG2(2), CHG3(4), CD6(9), COS-II(1)
2–3 years	97	6 (6.19%, 2.30–12.98%)	CHG2(1), CHG3(1), CD6(3), FJG-1(1)
≥3 years	33	0 (0%, 0.00–10.58%)	
Total	539	42 (7.79%, 5.67–10.39%)	CHG1(1), CHG2(3), CHG3(8), CHG5(3), BEB6(4), CD6(20), COS-II(1), FJG-1(2)

**Table 4:** *E. bieneusi* genotypes of different gender in Fujian province.

Gender	Sample Number	Infection Rate (95% CI)	Assemblages
Male	161	15 (9.32%, 5.31–14.90%)	CHG1(1), CHG2(1), CHG3(1), CHG5(3), BEB6(1), CD6(7), COS-II(1)
Female	378	27 (7.14%, 4.76–10.22%)	CHG2(2), CHG3(7), BEB6(3), CD6(13), FJG-1(2)
Total	539	42 (7.79%, 5.67–10.39%)	CHG1(1), CHG2(3), CHG3(8), CHG5(3), BEB6(4), CD6(20), COS-II(1), FJG-1(2)

**Table 5:** MLST types of *E. bieneusi* in black goat.

Sample Number	ITS Genotyping	Genotype			MLGS
		MS1	MS4	MS7	
Ly1	CHG5	TypeI	TypeI	TypeI	MLGI (1)
Ly71	CHG1	TypeII	TypeII	TypeII	MLGII (1)
pt42	CD6	TypeI	TypeIII	TypeII	MLGIII (1)
Pt44	CD6	TypeI	TypeIV	TypeII	MLGIV (1)
Pt51	CD6	TypeI	TypeV	TypeI	MLGV (1)
Zz47	BEB6	TypeI	TypeVI	TypeI	MLGVI (1)
Zz59	CHG2	TypeIII	TypeIII	TypeI	MLGVII (1)
Zz76	CHG2	TypeIV	TypeIII	TypeI	MLGVIII (1)
Fz15	CHG3	TypeI	TypeVII	TypeII	MLGIX (1)
Sm7	CHG2	TypeV	TypeIII	TypeII	MLGX (1)

Note: Types I–VII represent different allele types identified at each microsatellite (MS1, MS7) and minisatellite (MS4) locus based on sequence polymorphisms compared to reference sequence. Types were assigned sequentially as new alleles were detected.

## Data Availability

The original contributions presented in this study are included in the article/Supplementary Materials. Further inquiries can be directed to the corresponding author.

## Supplementary Materials

The following supporting information can be downloaded at https://www.mdpi.com/article/10.3390/pathogens15030299/s1, Figure S1. Partial PCR amplification products of the ITS gene resolved by agarose gel electrophoresis; Figure S2. Electrophoresis of partial PCR amplification of MS1 sites in *E. bieneusi*. M: DL2000 DNA marker; Figure S3. Electrophoresis of partial PCR amplification of MS4 sites in *E. bieneusi*. M: DL2000 DNA marker; Figure S4. Electrophoresis of partial PCR amplification of MS7 sites in *E. bieneusi*. M: DL2000 DNA marker; Table S1. The primers of *Enterocytozoon bieneusi* used in this study.
