# Supplementary material for: Occurrence and Multi-Locus Genotyping of Enterocytozoon bieneusi in Black Goats from Fujian Province, China
Source: Pathogens. 2026 Mar 10;15(3):299. doi: 10.3390/pathogens15030299 (PMC13028804; doi:10.3390/pathogens15030299)
Supplement: Supplementary file 1 [file pathogens-15-00299-s001.zip › pathogens-4164509-supplementary.pdf]

Supplementary Materials

Supplementary Figures

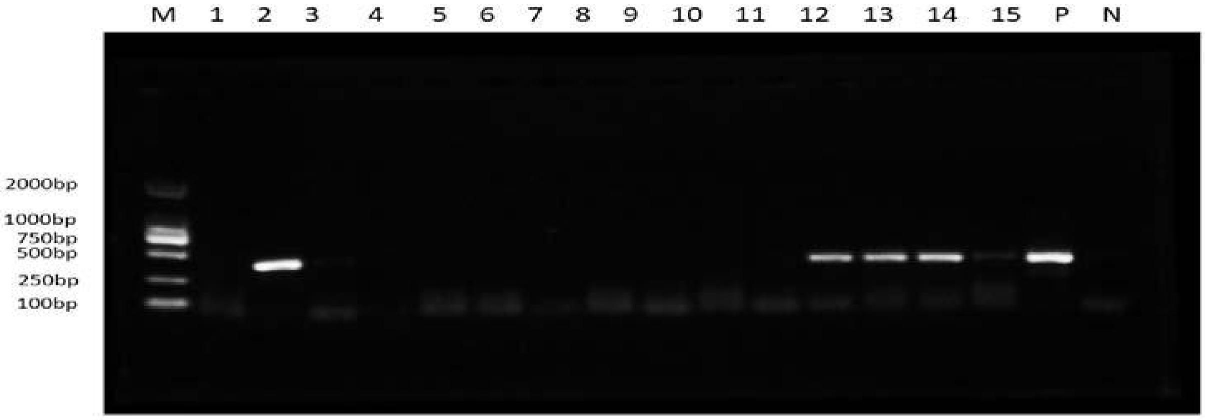

Figure S1. Partial PCR amplification products of the ITS gene resolved by agarose gel electrophoresis. M: DL2000 DNA marker; Lines 1-15: samples; P: Positive DNA; N: Negative DNA.

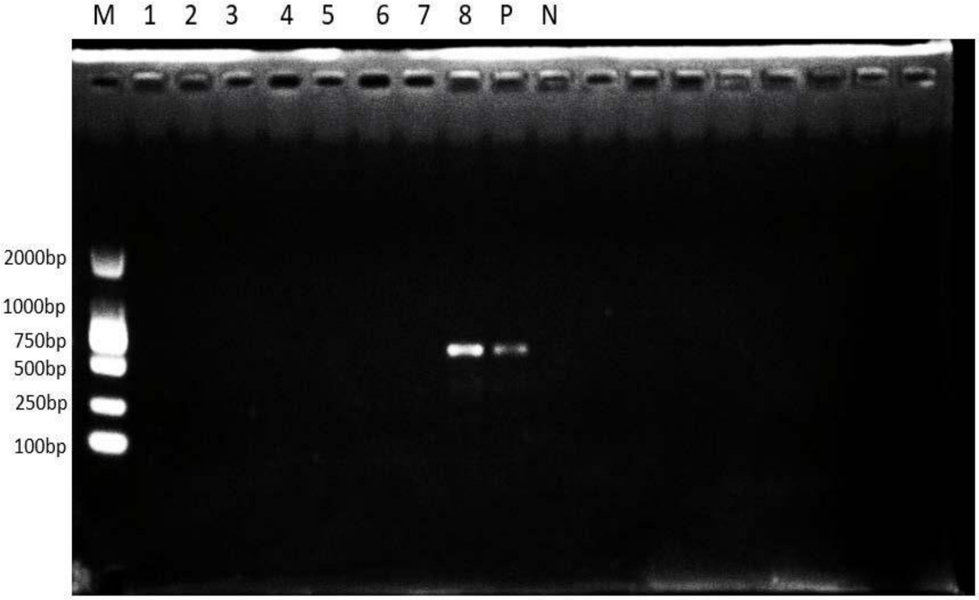

Figure S2. Electrophoresis of partial PCR amplification of MS1 sites in *E. bieneusi*. M: DL2000 DNA marker. Lines 1-8: samples; P: Positive DNA; N: Negative DNA.

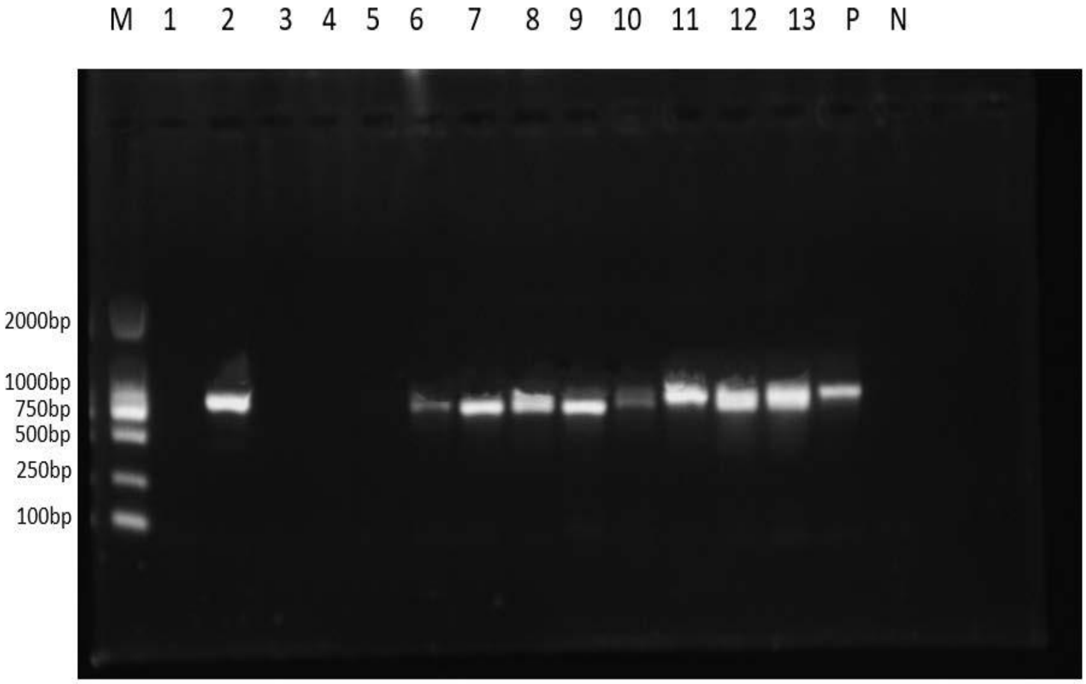

Figure S3. Electrophoresis of partial PCR amplification of MS4 sites in *E. bieneusi*. M: DL2000 DNA marker. Lines 1-13: samples; P: Positive DNA; N: Negative DNA.

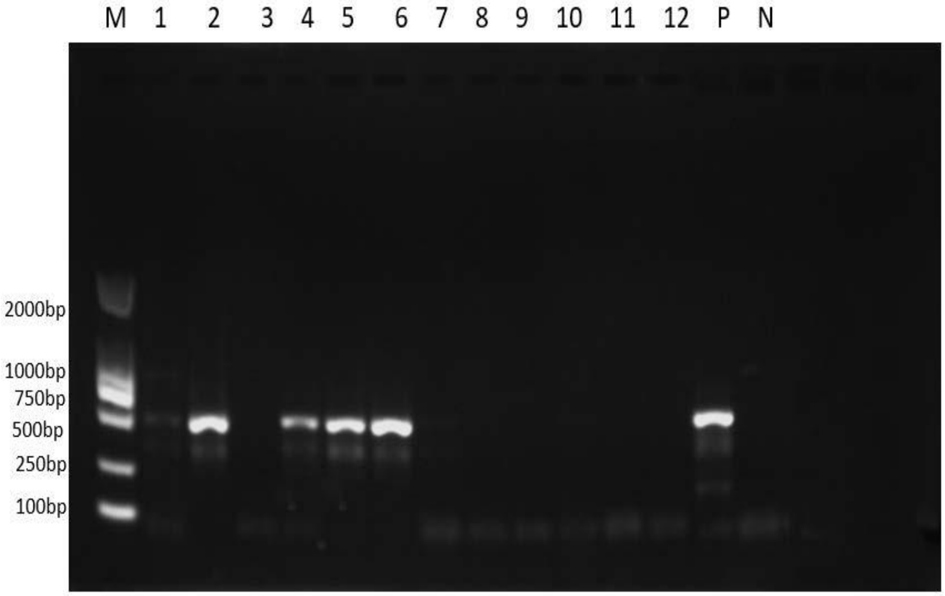

Figure S4. Electrophoresis of partial PCR amplification of MS7 sites in *E. bieneusi*. M: DL2000 DNA marker. Lines 1-12: samples; P: Positive DNA; N: Negative DNA.

Supplementary Table

Table S1. The primers of *Enterocytozoon bieneusi* used in this study.

Locus	Sequences (5′-3′)	Length(bp)	Annealing Temperature（℃）
ITS	F1: 5′-GGTCATAGGGATGAAGAG-3′<br>R1: 5′-TTCGAGTTCTTTCGCGCTC-3′	410 bp	57℃
	F2: 5′-GCTCTGAATATCTATGGCT-3′<br>R2: 5′-ATCGCCGACGGATCCAAGTG-3′	389 bp	55℃
MS1	F1: 5′-CAAGTTGCAAGTTCAGTGTTTGA-3′<br>R1: 5′-GATGAATATGCATCCATTGATGTT-3′	843 bp	58℃
	F2: 5′-TTGTAAATCGACCAAATGTGCTAT-3′<br>R2: 5′- GGACATAAACCACTAATTAATGTAAC-3′	675 bp	58℃
MS3	F1: 5′-CAAGCACTGTGGTTACTGTT-3′<br>R1: 5′-AAGTTAGGGCATTTAATAAAATTA-3′	702 bp	55℃
	F2: 5′-GTTCAAGTAATTGATACCAGTCT-3′<br>R2: 5′-CTCATTGAATCTAAATGTGTATAA-3′	537 bp	55℃
MS4	F1: 5′-GCATATCGTCTCATAGGAACA-3′<br>R1: 5′-GTTCATGGTTATTAATTCCAGAA-3′	1066 bp	55℃
	F2: 5′-CGAAGTGTACTACATGTCTCT-3′<br>R2: 5′-GGACTTTAATAAGTTACCTATAGT-3′	885 bp	55℃
MS7	F1: 5′-GTTGATCGTCCAGATGGAATT-3′<br>R1: 5′-GACTATCAGTATTACTGATTATAT-3′	684 bp	55℃
	F2: 5′-CAATAGTAAAGGAAGATGGTCA-3′<br>R2: 5′-CGTCGCTTTGTTTCATAATCTT-3′	471 bp	55℃
